# High Prevalence of Plasmid-Mediated Quinolone Resistance and IncQ Plasmids Carrying *qnrS2* Gene in Bacteria from Rivers near Hospitals and Aquaculture in China

**DOI:** 10.1371/journal.pone.0159418

**Published:** 2016-07-18

**Authors:** Yanping Wen, Xiaoying Pu, Wei Zheng, Guang Hu

**Affiliations:** 1 Microbiology Laboratory, Hangzhou Center for Disease Control and Prevention, Hangzhou, Zhejiang, People’s Republic of China; 2 Department of Landscape Architecture, School of Civil Engineering and Architecture, Zhejiang Sci-Tech University, Hangzhou, Zhejiang, People’s Republic of China; Nankai University, CHINA

## Abstract

Effluents from hospital and aquaculture are considered important sources of quinolone resistance. However, little information is available on the impact of this effluent on nearby rivers. In this study, 188 ciprofloxacin-resistant bacterial isolates obtained from rivers near hospitals and aquaculture were screened for plasmid-mediated quinolone resistance (PMQR) genes. Species identification, antibiotic susceptibility testing, and PMQR gene transferability assessment were conducted for PMQR-positive bacteria. Representative *qnrS2-*encoding plasmids were subsequently sequenced using a primer-walking approach. In total, 44 isolates (23.4%) were positive for *qnr* genes (16 *qnrB2*, 3 *qnrS1*, and 25 *qnrS2*) and 32 isolates (17.0%) were positive for *aac(6′)-Ib-cr*. Other PMQR genes were not detected. The *qnrB2* and *aac(6′)-Ib-cr* genes had a higher prevalence in aquaculture samples than in hospital samples, and were significantly associated with *Enterobacteriaceae* (*p* < 0.05). In contrast, the prevalence of *qnrS2* was not site-related, but was significantly associated with *Aeromonas* spp. (*p* < 0.05). All PMQR isolates were resistant to three or more classes of antibiotics. Eleven *qnrS2*-harboring plasmids from *Aeromonas* spp., including a novel conjugative plasmid pHP18, were selected for sequencing. These plasmids were small in size (6,388–16,197 bp) and belonged to the IncQ or IncU plasmid family, with *qnrS2* being part of a mobile insertion cassette. Taken together, our findings suggest that aquaculture is a possible source for *aac(6′)-Ib-cr* and *qnrB2* dissemination, and demonstrate the ubiquity of *qnrS2* in aquatic environments. Finally, *Aeromonas* spp. served as vectors for *qnrS2* with the help of IncQ-type plasmids.

## Introduction

The dissemination of quinolone-resistant bacterial isolates has become a major problem in infection control worldwide. In Portugal, 23.8% of clinical *Escherichia coli* isolates were resistant to quinolones [1]. An even higher percentage was reported in the Shanghai area of China, where the frequency of ciprofloxacin resistance in *E*. *coli* has exceeded 50% since 1993 [2]. High-level resistance to quinolones is mainly associated with point mutations in the chromosomal genes *gyrA* and *parC*, which encode the quinolone targets DNA gyrase and topoisomerase IV, respectively. This chromosome-encoded resistance is transmitted vertically and thus believed to correlate with the phylogenetic lineage of the host. In contrast, plasmid-mediated quinolone resistance (PMQR) confers low-level resistance to quinolones by protection from drug targets and can be transferred horizontally among distantly related lineages [3]. This might favor the emergence of strains with higher resistance to quinolones because of chromosomal mutations. Three major mechanisms are involved in PMQR: (i) limiting quinolone inhibition by Qnr protein protection of drug targets; (ii) modification of the quinolone molecule by the variant aminoglycoside acetyltransferase Aac(6′)-Ib-cr; and (iii) the quinolone-specific efflux pump QepA. Therefore, PMQR might play a role in maintaining resistance levels in bacterial populations in the presence of sub-inhibitory concentrations of antibiotics [4].

Urban rivers provide an ideal setting for the acquisition and spread of PMQR because of continuous pollution by quinolones from anthropogenic sources [5–7], e.g., effluents from municipalities, hospitals, and aquaculture. Effluents from these sources may have a quinolone concentration high enough to exert selective pressure on environmental bacteria [8]. Additionally, effluents from these sources can contain a variety of quinolone-resistant pathogens and mobile genetic elements carrying PMQR genes [6, 9]. Discharge may alter biodiversity in an ecosystem, as well as the characteristic microbiota of water animals. Moreover, since several aquatic bacterial species have intrinsic *qnr* genes [10, 11], mixing with allochthonous species from different sources is likely to promote genetic exchange, which may exacerbate clinical drug resistance and reduce environmental quality.

There is a growing interest in exploring the emergence of PMQR in bacteria from hospital and aquaculture effluent [6, 9, 12, 13]. However, little information is available on the affected rivers, despite the quality of these rivers having a considerable influence on the health of nearby residents. Recent studies have indicated that wastewater treatment plants had a negligible effect on *qnrS* removal and resistance elimination [14–17]. The incomplete removal severely affects the receiving river, with *qnrS* found at higher concentration in downstream waters than in samples collected upstream from a discharge point [15].

The aim of this study was to investigate the prevalence and distribution of PMQR bacteria and their gene determinants in rivers that are likely affected by hospital and aquaculture effluent. The transferability and genetic environments of PMQR genes were also analyzed. Knowledge of the sources and mechanisms of PMQR dissemination in different environments can lead to the development of effective strategies to control antibiotic resistance and assess human health risk.

## Materials and Methods

### Bacterial Isolates

Since the sampling locations were urban rivers open to the public, and no endangered or protected species were involved, no specific permissions were required for this field study. Water samples were collected from 9 aquatic environments in Hangzhou, China, from November 2014 to October 2015. Of these, 4 were situated near hospitals or medical schools (H1–H4), 4 were near aquaculture (A1–A4), and 1 was water source conversation area and was considered as clean water (C). At each site, representative samples were collected in 500-mL glass bottles in triplicate at 3-month intervals. The particles were filtered through a sterile 0.22-μm membrane and inoculated onto Mueller-Hinton (MH) agar plates supplemented with 5 μg/mL ciprofloxacin. Individual colonies were picked up based on morphology. All experiments in this study were performed in parallel.

### Characterization of PMQR Bacteria and Their Gene Determinants

Total DNA was extracted using a simple boiling method. The *qnrA*, *qnrB*, and *qnrS* genes were screened using a multiplex PCR method as described previously [18]. The genes *qnrC*, *qnrD*, *aac(6′)-Ib-cr*, and *qepA* were also detected [19]. The 16S rRNA, *gyrB*, and *ropD* genes were amplified and sequenced for phylogenetic analysis [20].

### Transferability of PMQR Genes

Transferability of PMQR genes from the environmental isolates was tested, with *E*. *coli* J53 Azi^R^ and *E*. *coli* DH5α used as recipients for conjugation and transformation experiments [21], respectively. The transconjugants were screened on Luria-Bertani (LB) agar plates supplemented with sodium azide (100 μg/mL) plus ciprofloxacin (0.05 μg/mL). The transformants were selected on LB agar plates with ciprofloxacin (0.05 μg/mL). PCR was performed to identify the PMQR genes acquired by the *E*. *coli* transconjugants and transformants, using primers and conditions described elsewhere [18].

### Antibiotic Susceptibility Testing

Antibiotic susceptibility to amikacin, ampicillin, cephalothin, chloramphenicol, ciprofloxacin, erythromycin, nalidixic acid, streptomycin, trimethoprim/sulphamethoxazole, and tetracycline was assayed by the disc diffusion method, according to the guidelines of the Clinical and Laboratory Standards Institute (CLSI, 2012). *E*. *coli* ATCC 25922 was used as a control strain. The minimal inhibitory concentrations (MICs) of ciprofloxacin were determined using a broth microdilution method in accordance with CLSI guidelines.

### Plasmid Analysis

The *E*. *coli* transconjugants and transformants were grown overnight at 37°C in LB broth containing ciprofloxacin (0.05 μg/mL). Plasmid DNA was extracted using the Qiagen Plasmid Midi Kit (Qiagen Science Inc., MD, USA). Complete nucleotide sequences were determined using a primer walking strategy. The primers P1-F (5′-AACTCAATACCGTAGCAAT-3′) and P1-R (5′-TTTATGTCACGCCGAACT-3′), which target the *qnrS2* gene and read outward, were applied to long-range PCR. Thermal cycling conditions were as follows: 1 min at 94°C; 35 cycles of 15 s at 93°C, 30 s at 55°C, and 7 min at 72°C,followed by a 7 min extension at 72°C. Direct sequencing was carried out using ABI PRISM Big Dye Terminator Cycle Sequencing technology (Applied BioSystems, Foster City, USA). Results were analyzed using the software DNA Sequencing Analysis V5.1 (ABI). A sequence analysis was carried out using BLASTn, BLASTx, DNASTAR Lasergene 8.0, and the open reading frame (ORF) finder program (http://www.ncbi.nlm.nih.gov/gorf/gorf.html). The plasmids were named according to their hosts.

### Phylogenetic Analysis

Multiple sequence alignments were performed using ClustalW in MEGA 5.10 [22]. Phylogenetic trees were constructed using the neighbor-joining method, and the robustness was evaluated by bootstrap analyses based on 1000 resamplings.

### Statistics

Statistical analyses were performed using SPSS 22.0 for Windows (SPSS Inc., Chicago, IL). The prevalence of PMQR genes or antibiotic resistance phenotypes was compared among isolates from different origins or taxonomic groups using the chi-square test at a significance level of 0.05.

### Nucleotide Sequence Accession Numbers

Representative 16S rRNA, *gyrB*, and *rpoD* gene sequences were deposited in GenBank under accession numbers KU644678 to KU644707. The *aac(6′)-Ib*, *aac(6′)-Ib-cr*, and *qnr* gene sequences were submitted to GenBank with accession numbers KU644708 to KU644712. The complete nucleotide sequences of plasmids were deposited in GenBank under accession numbers KU644672 to KU644677.

## Results

### Prevalence of PMQR Genes in Aquatic Environments

In total, 188 ciprofloxacin-resistant bacterial isolates were obtained from rivers near hospitals (n = 85), aquaculture (n = 83), or clean water (n = 20). Through PCR-based screening, 56 environmental bacteria (29.8%, 56/188) were found to be PMQR positive (Table 1). *qnr* genes were detected in 44 isolates (23.4%, 44/188), including *qnrB2* in 16, *qnrS1* in 3, and *qnrS2* in 25 isolates. *aac(6′)-Ib-cr* genes were detected in 32 isolates (17.0%, 32/188). Other PMQR genes, including *qnrA*, *qnrC*, *qnrD*, and *qepA*, were not detected.

**Table 1:** Prevalence of plasmid-mediated quinolone resistance (PMQR) genes in water-borne environmental bacteria.

Sampling sites	Ciprofloxacin- resistant isolates (No.)	PMQR- positive isolates (No.)	Number of isolates positive for specific genes							Prevalence (%)			
			*qnrS1*	*qnrS2*	*aac(6')-Ib-cr*	*qnrB2*+ *aac(6')-Ib-cr*	*qnrS1*+ *aac(6')-Ib*	*qnrS2*+ *aac(6')-Ib*	*qnrS2*+ *aac(6')-Ib-cr*	*qnrS1*	*qnrS2*	*qnrB2*	*aac(6')-Ib-cr*
**Rivers near hospitals**	85	24	2	7	4	3	1	5	2	3.5	16.5	3.5	10.6
**Rivers near aquaculture**	83	31	0	4	8	13	0	4	2	0	12.0	15.7	27.7
**Clean water**	20	1	0	1	0	0	0	0	0	0	5	0	0
**Total**	188	56	2	12	12	16	1	9	4	2.1	12.8	8.5	17.0

The prevalence of PMQR in isolates from hospital-associated samples, aquaculture-associated samples, or clean water was 28.3% (24/85), 37.3% (31/83), and 5% (1/20), respectively. The prevalence of *qnrS1* and *qnrS2* genes was not associated with a specific sampling site (*p* > 0.05). The *aac(6′)-Ib-cr* and *qnrB2* genes showed a significantly higher prevalence in aquaculture samples than in hospital samples (*p* < 0.05), but their prevalence was not significantly different between hospital samples and clean water (*p* > 0.05).

**Fig 1:**
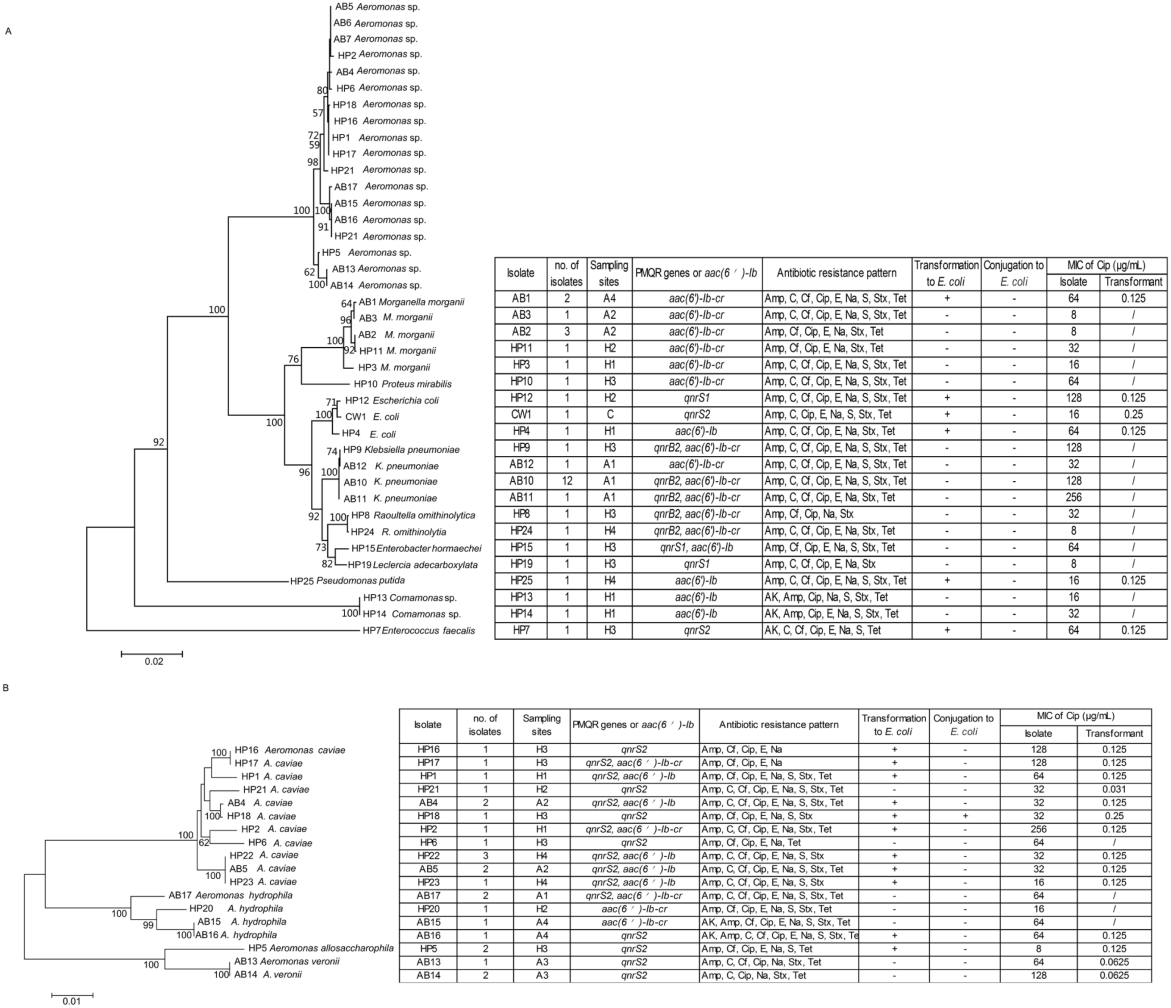
Neighbor-joining trees based on 16S rRNA gene sequences (A) and the concatenated nucleotide sequences of *gyrB* and *rpoD* genes (1342 bp) (B) from plasmid-mediated quinolone resistance (PMQR) isolates. Bootstrap values (≥50%) generated from 1000 replicates are indicated at branch points. The sampling sites, PMQR genes, antibiotic resistance, transferability of PMQR genes, and minimal inhibitory concentrations (MICs) of ciprofloxacin are indicated in tables. H1–H4, rivers near hospitals; A1–A4, rivers near aquaculture; C, clean water. AK, amikacin; Amp, ampicillin; C, chloramphenicol; Cf, cephalothin; Cip, ciprofloxacin; E, erythromycin; Na, nalidixic acid; S, streptomycin; Stx, trimethoprim/sulphamethoxazole; Tet, tetracycline.

Phylogenetic analyses based on the 16S rRNA, *gyrB*, and *ropD* genes showed that PMQR bacteria grouped into two main taxonomic groups, namely *Enterobacteriaceae* and *Aeromonadaceae* (Fig 1). The prevalence of some PMQR genes, including *qnrB2*, *qnrS2*, and *aac(6′)-Ib-cr*, was significantly associated with certain taxonomic groups (*p* < 0.05). Ninety-two percent of *qnrS2*-carrying strains were identified as belonging to *Aeromonadaceae*, whereas 100% of *qnrB2*- and 81.3% of *aac(6′)-Ib-cr*-carrying strains were identified as *Enterobacteriaceae*.

### Antibiotic Susceptibility of Environmental Isolates

**Fig 2:**
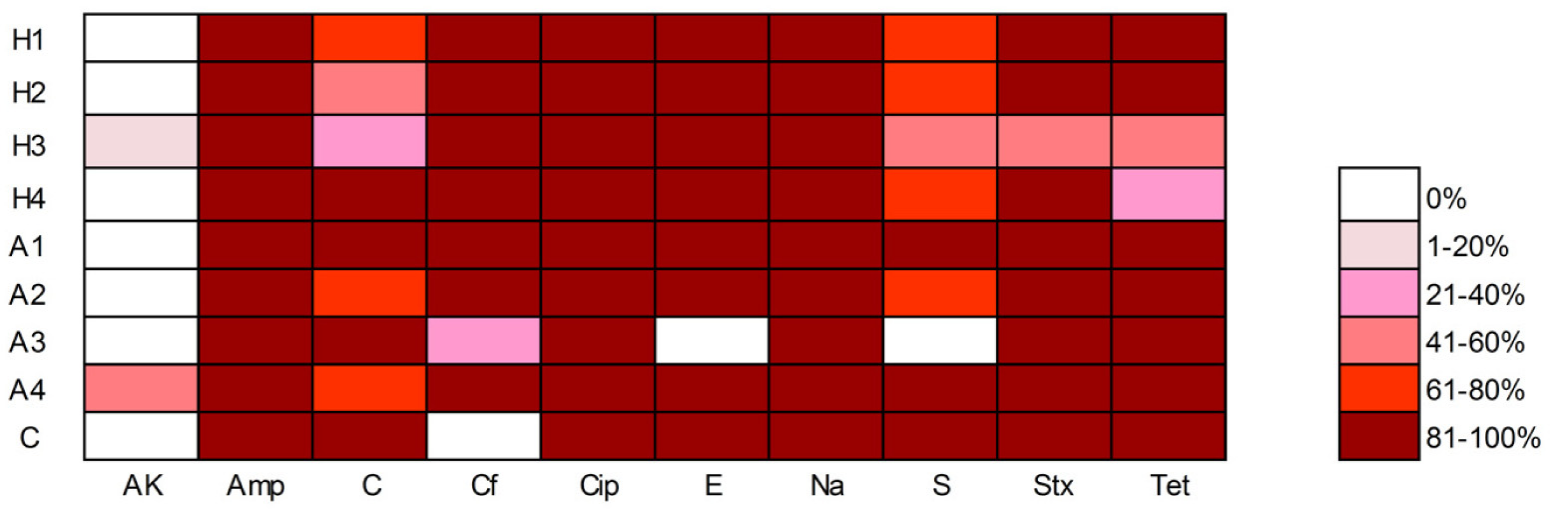
Heat map showing the association between antibiotic resistance patterns and sampling sites. H1–H4, rivers near hospitals; A1–A4, rivers near aquaculture; C, clean water. AK, amikacin; Amp, ampicillin; C, chloramphenicol; Cf, cephalothin; Cip, ciprofloxacin; E, erythromycin; Na, nalidixic acid; S, streptomycin; Stx, trimethoprim/sulphamethoxazole; Tet, tetracycline.

All PMQR isolates (n = 56) presented multidrug resistance (MDR), i.e., resistance to antibiotics belonging to three or more classes (Fig 1). The most commonly encountered resistances were to ciprofloxacin (100%, 56/56) and nalidixic acid (100%, 56/56), followed by ampicillin (98.2%, 55/56), cephalothin (94.6%, 53/56), erythromycin (92.9%, 52/56), trimethoprim/sulphamethoxazole (89.3%, 50/56), tetracycline (83.9%, 47/56), chloramphenicol (73.2%, 41/56), streptomycin (73.2%, 41/56), and amikacin (5.4%, 3/56) (Fig 2).

For most antibiotic classes, the prevalence of resistance did not significantly differ (*p* > 0.05) by sampling site. The exception was the significantly higher (*p* < 0.05) prevalence of resistance to chloramphenicol, erythromycin, and sulfamethoxazole/trimethoprim in isolates from aquaculture samples. The average MICs of ciprofloxacin against *Enterobacteriaceae* (82 μg/mL) and *Aeromonadaceae* (62 μg/mL) were not significantly different (*p* > 0.05).

### Transfer of PMQR Genes

Of the *Enterobacteriaceae* (n = 29), 4 strains (13.8%) were able to transfer PMQR genes to *E*. *coli* DH5α by transformation of [2 *aac(6′)-Ib-cr*, 1 *qnrS1*, and 1 *qnrS2*], but all failed to produce transconjugants. Of the *Aeromonadaceae* (n = 23), 16 strains (69.6%) were able to transfer PMQR genes to *E*. *coli* DH5α by transformation of [5 *qnrS2*, 9 *qnrS2* associated with *aac(6’)-Ib*, and 2 *qnrS2* associated with *aac(6′)-Ib-cr*]; however, only one transconjugant carrying *qnrS2* was obtained (Fig 1).

Overall, the rates of mobile *aac(6′)-Ib-cr*, *qnrB2*, *qnrS1*, and *qnrS2* genes were 12.5% (4/32), 0% (0/16), 33.3% (1/3), and 72.0% (18/25), respectively. The presence of PMQR genes increased the MICs of ciprofloxacin two- to eight-fold compared with those in the control *E*. *coli* recipients (Fig 1).

### Characterization of *qnrS2*-Harboring Plasmids in *Aeromonas* spp.

**Fig 3:**
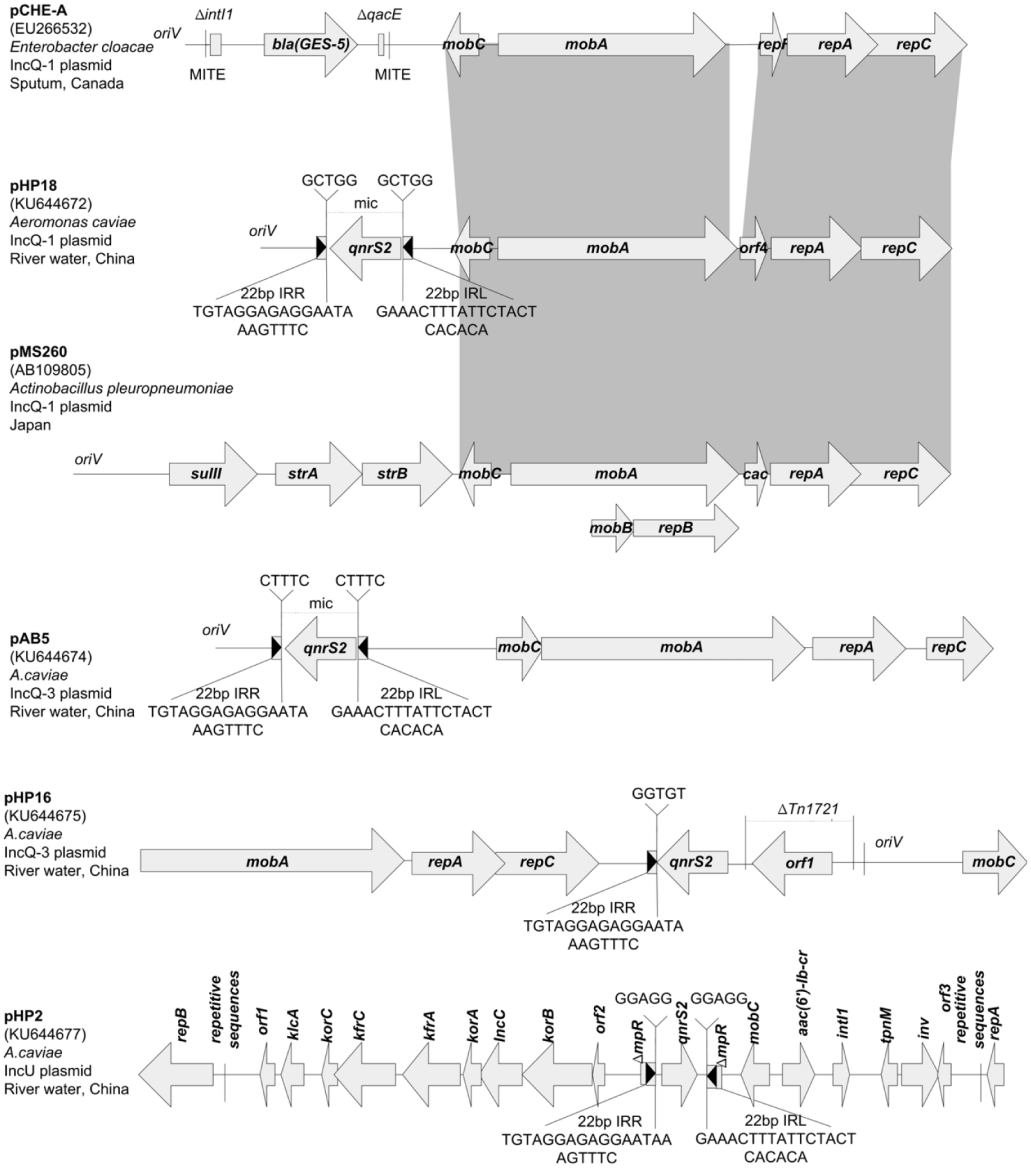
Linear maps of the *qnrS2*-positive plasmids. Open reading frames (ORFs) are shown as arrows indicating the direction of transcription. Inverted repeats (IRL, left inverted repeat; IRR, right inverted repeat) are shown in boxes with black arrows indicating the direction. Their lengths and sequences are shown below the structures. Direct repeats flanking the mobile insertion cassette (mic) are shown above the map. Homologous regions are indicated by gray shading. MITE, miniature inverted transposable element.

Eleven *E*. *coli* transformants derived from clonally unrelated *Aeromonas* strains were selected for plasmid analysis. These plasmids were small in size (6,388–8,663 bp) and belonged to the IncQ family except for an IncU plasmid, pHP2 (16,197 bp). All plasmids contained *qnrS2* inserted at either side of the *oriV* region (IncQ) or the gene *mpR* coding for a putative zinc-metalloprotease (IncU) in the form of a mobile insertion cassette (mic). The mic was bracketed by 22-bp imperfect inverted repeats and a 5-bp duplication of the target site (Fig 3), suggesting the acquisition of this structure by transposition. Based on genetic structure, three IncQ plasmid groups were identified.

**Fig 4:**
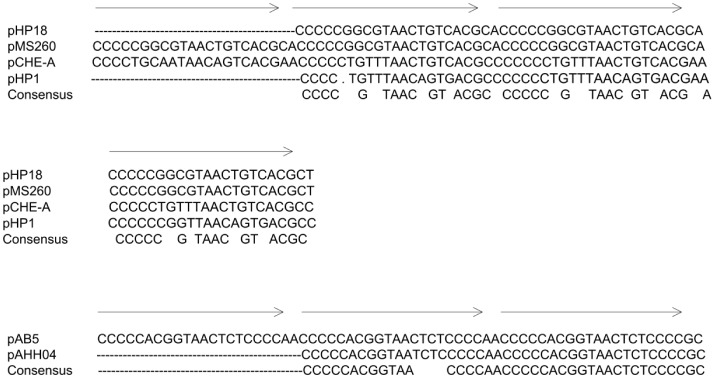
Nucleotide sequence alignment of the iterons within the *oriV* of the IncQ plasmids. Direct repeats are shown by arrows above the sequence. Consensus sequences between plasmids are shown.

**Fig 5:**
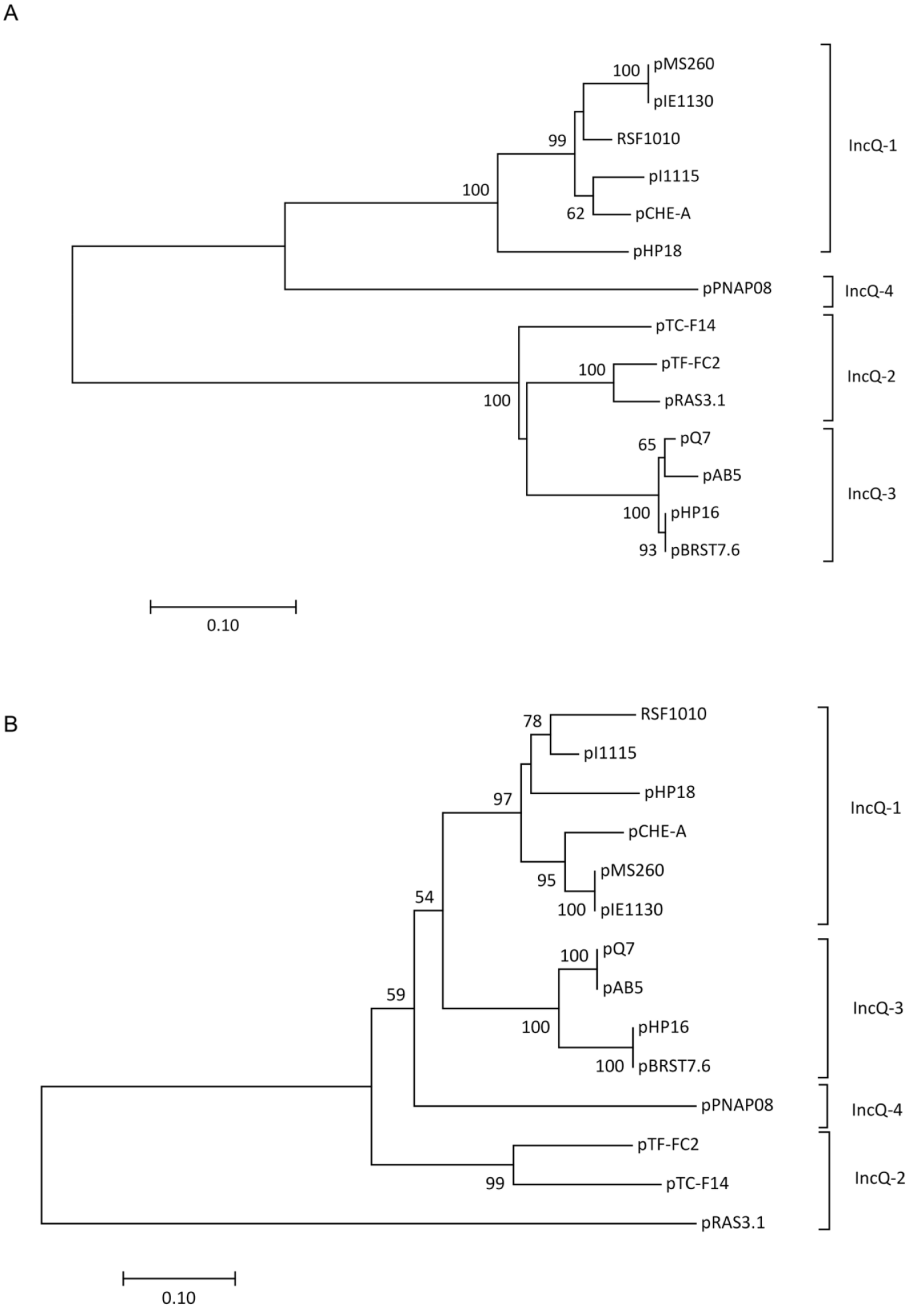
Neighbor-joining trees based on RepA helicase (A) and RepC iteron-binding protein (B) from the IncQ plasmids. Bootstrap values (≥50%) generated from 1000 replicates are indicated at branch points.

The first IncQ plasmid group was isolated from *Aeromonas caviae* and included two identical plasmids, pHP18 and pAB4, and one variant pHP1. Plasmid pHP18, the sole plasmid found in this study, which was capable of mobilization through conjugation, was 6,388 bp in size with a G+C content of 57%. It contained 6 ORFs: *repA* and *repC* genes for plasmid replication, *mobA* and *mobC* genes for plasmid mobilization, the *qnrS2* gene for quinolone resistance, and a repressor gene (Table 2). The *qnrS2* gene was 100% identical with its *Aeromonas* spp. counterpart. The plasmid backbone showed high similarity with two IncQ-1 plasmids, pCHE-A (66% coverage, 85% identity) and pMS260 (65% coverage, 80% identity). Plasmid pCHE-A originated from *Enterobacter cloacae* from Canada, and harbored the *bla*_GES-5_ gene [11], whereas pMS260 was a streptomycin and sulfonamide resistance-coding plasmid isolated from *Actinobacillus pleuropneumoniae* in Japan [23]. Despite sharing three common 22-bp iterons with pMS260, the backbone of pHP18 showed characteristics that were distinct from those of other IncQ-family plasmids, including entirely different A+T-rich and G+C-rich regions, and the absence of a highly conserved 15-bp region. A codon bias analysis indicated that the backbone genes of pHP18 had a strong preference for a G or C in the third position (72.8%), while only 39.3% of the codons of *qnrS2* had a G or C residue in that position. Compared with pHP18, pHP1 had an additional 22-bp iteron insertion within *oriV* (Fig 4). Phylogenetic analysis of the replication protein sequences showed that pHP18 and pHP1 grouped with the IncQ-1 family (Fig 5).

**Table 2:** Predicted open reading frames (*orfs*) of pHP18.

Coding region (start-end)	*orfs*/Genes	No. of amino acids	Function	% Amino acid identity with nearest phylogenetic relative (Accession No.)
**Complement 629–1285**	*orf*1/*qnrS2*	218	Quinolone resistance protein	100%, QnrS2 coded by pBRST7.6 of *Aeromonas hydrophila* (YP_002221301)
**Complement 1769–2107**	*orf*2/*mobC*	112	Mobilization protein MobC	60%, mobilization protein of *Haemophilus ducreyi* (AKO40377)
**2184–4400**	*orf*3/*mobA*	738	Mobilization protein MobA	71%, mobilization protein of *Escherichia coli* (END87849)
**4426–4683**	*orf*4	85	Repressor protein	79%, repressor of *Klebsiella pneumonia* (KPR93843)
**4714–5556**	*orf*5/*repA*	280	Plasmid replication-related protein RepA	87%, RepA of *Enterobacter cloacae* (YP_002563152)
**5546–6388**	*orf*6/*repC*	280	Plasmid replication-related protein RepC	90%, RepC coded by pM3446F of *Actinobacillus pleuropneumoniae* (AKM21213)

The second IncQ plasmid group included three identical plasmids from *A*. *caviae*. The representative plasmid pAB5 was 7,212 bp in size, had a G+C content of 60%, and contained 5 ORFs: *repA*, *repC*, *mobA*, *mobC*, and *qnrS2*. pAB5 showed a high degree of similarity (99.9%) with the *qnrS2-*carrying plasmid pAHH04 from *A*. *hydrophila*, except for the iteron region [24](Fig 4), and belonged to the IncQ-3 group (Fig 5).

The third IncQ plasmid group was widely distributed among the *Aeromonas* species, including *A*. *caviae*, *A*. *hydrophila*, and *A*. *allosaccharophila*. This group consisted of three identical plasmids (pHP16, pHP17, and pAB16) and one variant pHP5. Plasmid pHP16 was 8,213 bp in size, had a G+C content of 59%, and belonged to the IncQ-3 group (Fig 5). It contained 6 ORFs: *repA*, *repC*, *mobA*, *mobC*, *qnrS2*, and a methyl-accepting chemotaxis protein-encoding gene (*orf1*). Plasmid pHP16 showed a sequence nearly identical to that of plasmids pHP5, pGNB2, and pBRST7.6 [25, 26], except for a variable region located between *qnrS2* and *oriV* (nt 7367–8013 bp in pHP16) that resulted from different truncation patterns in Tn1721.

The plasmid pHP2, harboring the *qnrS2* and *aac(6′)-Ib-cr* genes in *A*. *caviae*, had a typical IncU backbone for plasmid replication and maintenance functions (Fig 3). It was 16,197 bp in size, with an average G+C content of 55%. *qnrS2* was inserted into *mpR* as a mic. *aac(6′)-Ib-cr*, located on an incomplete class 1 integron, was inserted into the gene *nic*, encoding VirD2 relaxase. pHP2 was highly related to the PMQR-carrying IncU plasmid pAH6 (98% coverage, 100% identity) and pAC3 (100% coverage, 99% identity) found in *Aeromonas* spp. from Czech Republic and Korea [27]. The divergence was mainly due to different repetitive sequences surrounding the *rep* genes. In pHP2, the repetitive sequences located upstream of *repA* (319 bp) and downstream of *repB* (739 bp) were organized as (r3r1)_3_r3 and (r1)_8_r2, respectively. The corresponding sequences in pAH6 were organized as (r3r1r3)_2_ and (r1)_8_r2, whereas in pAC3 the counterparts were organized as (r3r1)_3_r3 and r1(partial)r1(partial)r1r2. Furthermore, the left inverted repeat of mic in pHP2, as well as a partial truncated region of *mpR*, was missing in pAH6. This difference may have resulted from plasmid rearrangements subsequent to the acquisition of the *qnrS2*-carrying mic.

## Discussion

The PMQR determinants have been identified in a number of environmental bacteria worldwide. Their prevalence appears to vary considerably depending on the selection criteria of studied strains, ranging from 16.7 to up to 58.0% [9, 28]. In this study, the prevalence of PMQR genes in isolates from rivers was 29.8%, with *aac(6′)-Ib-cr* and *qnrS2* as the predominant genes. This conclusion was supported by previous studies demonstrating the frequent occurrences of these genes in environmental isolates [9, 15, 28]. The *qnrS*-type genes seem to be the most commonly identified acquired *qnr* genes in the environment [4]. They have been mainly identified from waterborne species, and in particular *Aeromonas* spp. [29–31]. *aac(6′)-Ib-cr* is widespread geographically and stable over time [32]. It has often been more common than *qnr* alleles. The occurrence of *qepA* is quite limited among environmental isolates, but in animals might be significant, with a strong association with *rmtB* genes [33]. Noticeably, some studies identified a high prevalence of other PMQR genes such as *qnrD* or *qnrB* in aquatic environments [2, 28]. Factors, such as bacterial species and microbial habitat, may play important roles in the development and spread of antibiotic resistance.

In this study, the prevalence of the genes *aac(6′)-Ib-cr* and *qnrB2* was significantly higher in aquaculture samples than in hospital samples. However, there was no significant difference between hospital samples and clean water. This suggests a site-related dissemination route and a non-clinical origin for the two genes. Rivers near aquaculture may serve as a source of *aac(6′)-Ib-cr* and *qnrB2* genes in the urban water cycle, as indicated by other studies focused on fish farms [34, 35]. A site-related prevalence may be related to a given habitat, where the success of resistance acquisition depends on the fitness of the target bacterium (receivers of horizontal gene transfer) [14]. In agreement with this observation, *aac(6′)-Ib-cr* and *qnrB2* genes were mainly detected in a specific taxonomic group, *Enterobacteriaceae*. There may be environmental conditions or selective pressures imposed in rivers near aquaculture that contribute to this site-related dissemination pattern.

Unlike *aac(6′)-Ib-cr* and *qnrB2*, the prevalence of *qnrS2* was not site-related; rather, it was species-related. The fact that *qnrS2* was mainly identified from *Aeromonas* spp., typical inhabitants of aquatic environments, suggested a non-clinical source for this gene in bacteria from urban rivers. This conclusion is supported by previous studies demonstrating the frequent occurrence of *qnrS2* in municipal wastewater and rare detection in hospital effluent [9, 14, 16]. The ubiquity and genomic plasticity of *Aeromonas* spp. may contribute to the homogeneous distribution of *qnrS2* in water.

All PMQR bacteria examined showed resistance to more than three classes of antibiotics. In *Enterobacteriaceae*, multidrug resistance may be related to the high prevalence of *aac(6′)-Ib-cr*, which confers resistance to kanamycin, tobramycin, netilmicin, amikacin, and ciprofloxacin, and is associated with an MDR phenotype [6, 36]. Moreover, *qnr* genes have been found to exist in many extended-spectrum beta-lactamase- and AmpC-producing *Enterobacteriaceae* [37–39], suggesting their co-selection with other resistance elements. In *Aeromonadaceae*, because of their intrinsic resistance to some beta-lactams and susceptibility to antibiotic resistance acquisition [9], *Aeromonas* spp. are frequently multidrug resistant [16, 17]. The significantly higher prevalence of resistance to chloramphenicol, erythromycin, and sulfamethoxazole/trimethoprim in isolates from aquaculture samples may be associated with the wide range of antibiotics used in aquaculture [40]. High rates of resistance to the older, heavily used antibiotics (chloramphenicol, sulfonamides, and tetracyclines) have frequently been detected in fish food and fish pathogens such as *Aeromonas salmonicida* [34, 35, 41].

The *qnrS2* genes from *Aeromonas* spp. were often identified in IncU, IncQ, and ColE plasmids [24, 25, 27, 42]. In this study, IncQ plasmids were highly prevalent (90.9%). The wide distribution of IncQ plasmids carrying PMQR genes can be attributed to several mechanisms, including their ability to readily mobilize and replicate in a broad range of hosts, their low molecular weight that minimizes metabolic load, and their high copy numbers that ensure stability [43]. The ubiquity of *Aeromonas* species may have facilitated evolution by recombination of IncQ plasmids with a large number of genetic elements or resistance gene insertions.

Among *qnrS2*-carrying plasmids in *Aeromonas* spp., pHP18 is the first identified plasmid that can be mobilized by conjugation. However, it did not carry the mating pair formation (Mpf) components essential for efficient transfer by conjugation. A larger conjugative plasmid may be co-resident with pHP18, providing it with the Mpf components. A resistance gene reservoir not only allows resistant genes to stably exist, but also facilitates transfer of these genes to other species in a natural state. Our findings highlighted the important role of *Aeromonas* species as a PMQR gene reservoir. Plasmid pHP18 shows >80% identity with plasmids from *A*. *pleuropneumoniae* and *E*. *cloacae* in the backbone region, suggesting a possible origin from bacterial pathogens of humans or animals. The codon bias analysis indicated that *qnrS2* was likely inserted after a functional replicon had evolved in IncQ family plasmids.

In addition to the accessory genes, three 22-bp iterons in IncQ plasmids were subject to insertion or mutation, as shown in the pHP18 and pAB5 variants. Iterons were the major incompatibility determinants in IncQ plasmids, so point mutations within the three 20-bp conserved repeats of iterons could result in an inability or significantly reduced ability to exert incompatibility, initiate replication, or transfer genes by conjugation [43]. Meanwhile, the iteron number had a negative effect on plasmid copy number, as well as the antibiotic resistance level, and imposed metabolic burden [44]. Hence, variations in the iteron sequence may reflect the need for balance between high antibiotic resistance and a stable existence [45]. This may explain why pHP18 conferred a higher level of quinolone resistance to *E*. *coli* recipients than pHP1 did.

In IncU plasmids, a fragment encompassing *repB* with r1/r2 repetitive regions was essential for minireplicon functionality [46]. Other repetitive regions seemed to be a hotspot for sequence elimination, as shown in the pHP2 variants. Plasmid pHP2 is one of the smallest IncU plasmids identified so far. Recently, a smaller IncU plasmid pPA-2 (7,995 bp) was identified in *Pseudomonas aeruginosa* clinical isolates [47]. Both these plasmids have an elimination of conjugative transfer region. Despite loss of conjugative abilities, small IncU plasmids can be efficiently transferred in aquatic environments by transformation, due to the general ability of *Aeromonas* environmental isolates to acquire free DNA [48].

Overall, this study suggested aquaculture as a possible source of *aac(6′)-Ib-cr* and *qnrB2* in aquatic environments. *Enterobacteriaceae*, monitored in water quality controls, were important hosts of these two genes. The ubiquitous bacteria, *Aeromonas* spp., served as vectors for *qnrS2* with the help of IncQ-type plasmids. Evidence for water-human transmission of *Aeromonas* species was available [49, 50], and a *qnrS*-containing plasmid was identified in an *Aeromonas* sp. clinical isolate [51]. Given to the patterns of acquired antimicrobial resistance, more attentions should be paid to the antibiotic resistance surveillance of both clinical and environmental *Aeromonas* isolates.
